# Supplementary material for: Mechanisms governing the pioneering and redistribution capabilities of the non-classical pioneer PU.1
Source: Nat Commun. 2020 Jan 21;11:402. doi: 10.1038/s41467-019-13960-2 (PMC6972792; doi:10.1038/s41467-019-13960-2)
Supplement: Supplementary file 7 — Source data [file 41467_2019_13960_MOESM7_ESM.zip › Source_Data/FigureS2/FigureS2B_MotifScanOutput/KmeansCluster3/homerResults/motif28.similar.html]

motif28

## Information for motif28

C
T
A
G
C
T
G
A
G
A
C
T
G
A
C
T
A
T
C
G
C
T
A
G
C
T
G
A
A
G
C
T
A
G
T
C
C
T
G
A
A
G
C
T
  
Reverse Opposite:  

T
C
G
A
A
G
C
T
C
T
A
G
C
T
G
A
A
G
C
T
G
A
T
C
T
A
G
C
C
G
T
A
C
T
G
A
A
G
C
T
G
A
T
C
  

|  |  |
| --- | --- |
| p-value: | 1e-34 |
| log p-value: | -7.983e+01 |
| Information Content per bp: | 1.871 |
| Number of Target Sequences with motif | 77.0 |
| Percentage of Target Sequences with motif | 1.61% |
| Number of Background Sequences with motif | 79.7 |
| Percentage of Background Sequences with motif | 0.19% |
| Average Position of motif in Targets | 101.3 +/- 73.2bp |
| Average Position of motif in Background | 103.6 +/- 63.2bp |
| Strand Bias (log2 ratio + to - strand density) | 0.0 |
| Multiplicity (# of sites on avg that occur together) | 1.00 |
| Motif File: | file (matrix) reverse opposite |

### Similar de novo motifs found

|  |  |  |  |  |  |  |  |
| --- | --- | --- | --- | --- | --- | --- | --- |
| Rank | Match Score | Redundant Motif | P-value | log P-value | % of Targets | % of Background | Motif file |
| 1 | 0.803 | A C G T C T A G C T G A A G C T A G C T A T G C C T G A C T G A A G C T A G T C C G T A A G T C | 1e-26 | -60.080867 | 1.72% | 0.33% | motif file (matrix) |
| 2 | 0.909 | C G T A A G C T A G C T A C T G A C T G C G T A A C G T A G T C C T G A | 1e-15 | -36.568252 | 1.32% | 0.33% | motif file (matrix) |
